# The role of epigenetics in multi‐generational transmission of asthma: An NIAID workshop report‐based narrative review

**DOI:** 10.1111/cea.14223

**Published:** 2022-10-06

**Authors:** Lisa M. Wheatley, John W. Holloway, Cecilie Svanes, Malcolm R. Sears, Carrie Breton, Alexey V. Fedulov, Eric Nilsson, Donata Vercelli, Hongmei Zhang, Alkis Togias, Syed Hasan Arshad

**Affiliations:** ^1^ National Institute of Allergy and Infectious Disease National Institutes of Health Bethesda Maryland USA; ^2^ Faculty of Medicine, Human Development and Health University of Southampton Southampton UK; ^3^ Department of Global Public Health and Primary Care University of Bergen Bergen Norway; ^4^ McMaster University Hamilton Ontario Canada; ^5^ University of Southern California Los Angeles California USA; ^6^ Warren Alpert Medical School of Brown University, Rhode Island Hospital Providence Rhode Island USA; ^7^ Washington State University Pullman Pullman Washington USA; ^8^ University of Arizona Tucson Arizona USA; ^9^ Division of Epidemiology, Biostatistics and Environmental Health, School of Public Health University of Memphis Memphis Tennessee USA; ^10^ Clinical and Experimental Sciences, Faculty of Medicine University of Southampton Southampton UK; ^11^ The David Hide Asthma and Allergy Centre St Mary's Hospital Newport UK

## Abstract

There is mounting evidence that environmental exposures can result in effects on health that can be transmitted across generations, without the need for a direct exposure to the original factor, for example, the effect of grandparental smoking on grandchildren. Hence, an individual's health should be investigated with the knowledge of cross‐generational influences. Epigenetic factors are molecular factors or processes that regulate genome activity and may impact cross‐generational effects. Epigenetic transgenerational inheritance has been demonstrated in plants and animals, but the presence and extent of this process in humans are currently being investigated. Experimental data in animals support transmission of asthma risk across generations from a single exposure to the deleterious factor and suggest that the nature of this transmission is in part due to changes in DNA methylation, the most studied epigenetic process. The association of father's prepuberty exposure with offspring risk of asthma and lung function deficit may also be mediated by epigenetic processes. Multi‐generational birth cohorts are ideal to investigate the presence and impact of transfer of disease susceptibility across generations and underlying mechanisms. However, multi‐generational studies require recruitment and assessment of participants over several decades. Investigation of adult multi‐generation cohorts is less resource intensive but run the risk of recall bias. Statistical analysis is challenging given varying degrees of longitudinal and hierarchical data but path analyses, structural equation modelling and multilevel modelling can be employed, and directed networks addressing longitudinal effects deserve exploration as an effort to study causal pathways.

## Graphical abstract

Parental and grandparental exposures influence risk of asthma in their offspring, in part mediated by epigenetic transfer of information across generations. An improved understanding of these effects will help to better estimate the true morbidity and mortality resulting from environmental exposures, and subsequently to develop concepts for prevention.

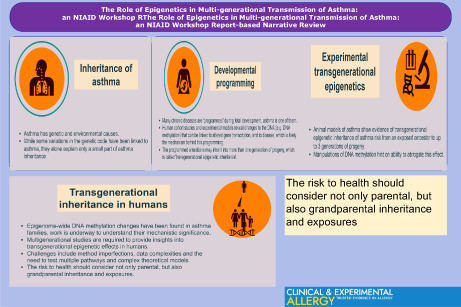

**Key messages**

- The risk to health should consider not only parental but also grandparental inheritance and exposures.
- Epigenetic processes might explain transgenerational effects, persisting in the absence of a direct environmental exposure.
- Multi‐generational studies are required to provide insights into transgenerational epigenetic effects in human.

## 1 INTRODUCTION

This review article is based on a workshop organized by the National Institute of Allergy and Infectious Diseases to bring together researchers interested in transgenerational effects focusing primarily, but not exclusively, on epigenetic transfer of information across generations. The specific objectives were to review and summarize the current status of knowledge for transgenerational effects in asthma and lung function, consider if epigenetic mechanisms explain this effect and identify major knowledge gaps and obstacles in furthering this field leading to recommendations for future research. The workshop focused on asthma, but the phenomenon has wider implications to other heritable chronic diseases such as diabetes and obesity.

## 2 THE INHERITANCE OF ALLERGIC DISEASE

The most frequently identified predictor of asthma is a family history of asthma or atopy.^1^ However, multi‐generational effects may reflect a number of mechanisms.^2^ These include a shared environment, genetic inheritance including genetic effects on the epigenome such as methylation quantitative trait loci and epigenetic inheritance. Exposures such as smoking, diet, occupation, microbiome, air pollution and farming environments are often shared among family members across generations, meaning that multi‐generational disease may not necessarily mean inherited disease.^3^, ^4^ Genetic inheritance is often assumed rather than proved. However, polygenic risk scores such as that developed based on a published asthma Genome‐Wide Association Study in the Dunedin (New Zealand) longitudinal development study^5^, ^6^, ^7^ do show that the likelihood of life‐course persistent asthma is, in part, related to inherited genetic factors. Epigenetic changes in cells/tissues represent the accumulated impacts of environmental exposures, life changes (developmental stages) and genetic variants.^8^ The role of epigenetics in asthma transmission between generations is thus of great interest and the focus of this review.

## 3 EPIGENETIC TRANSFER OF INFORMATION

Epigenetic factors are molecular factors or processes that regulate genome activity, independent of DNA sequence, and that are mitotically and/or meiotically stable.^9^ These factors include DNA methylation, histone modifications, non‐coding RNAs and chromatin structure which controls the accessibility of DNA regions or loops functional DNA domains into proximity to each other. Epigenetic processes preside over the maintenance and termination of gene expression, and the remodelling of chromatin architecture is critical to prepare genes for regulated transcription.

**FIGURE 1:**
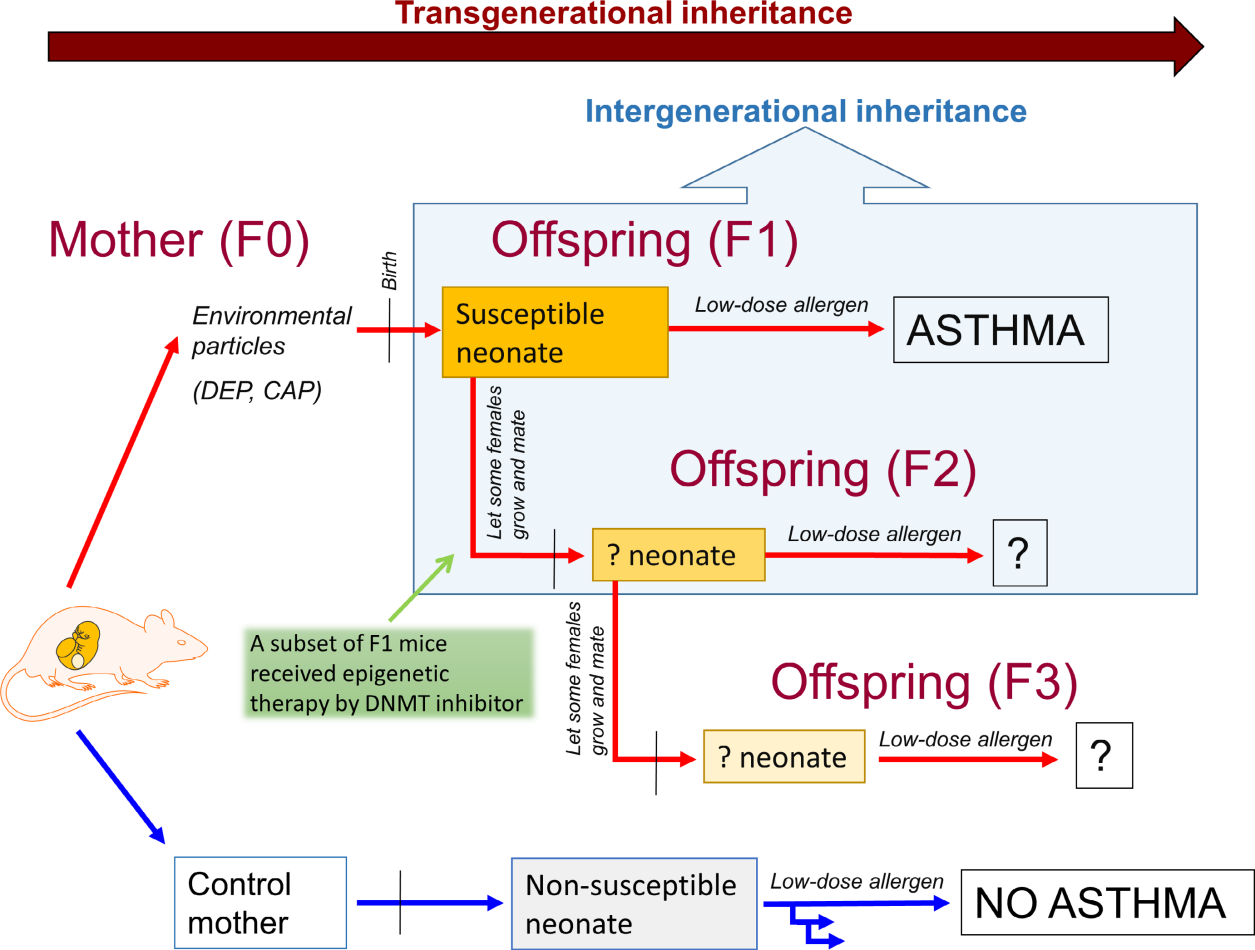
Transgenerational inheritance of disease risk: evidence from animal models. Gestational exposure of mice to an environmental trigger, in this case, diesel exhaust particles (DEP) or concentrated urban air particles (CAP), leads to increased asthma risk in up to 3 generations of offspring. It is important to distinguish intergenerational inheritance from transgenerational because in a gestational exposure model not only the pregnant F0 ancestor is directly exposed to the trigger, but also the fetus; moreover inside the F1 fetus there are predecessors of gametes that will give rise to F2 progeny which are theoretically also directly exposed. In the paternal line only the predecessors of gametes that form the F1 are directly exposed and effects that persist to the F2 can be considered transgenerational

In this report, intergenerational inheritance refers to maternal exposure having a direct effect on the developing fetus (and potentially on the germ line of the fetus), while transgenerational inheritance refers to the effect on subsequent generations persisting in the absence of a direct environmental exposure.^10^ If a pregnant female (F0 generation) is exposed to an environmental toxicant, then the embryo(s) she carries (generation F1), as well as the developing germ cells within those embryos that will form the F2 generation, are directly exposed. Therefore, the first generation in which one can observe transgenerational effects after exposure of a pregnant female is the F3 generation. Where a male parent or non‐pregnant female is exposed to an environmental toxicant, only the germ cells that will form the F1 generation are directly exposed, and therefore the first generation in which one can observe transgenerational effects is the F2 generation (Figure 1).

### 3.1 Epigenetic transgenerational inheritance

For environmentally induced epigenetic changes to be inherited, an altered epigenome must be present in germ cells (sperm or eggs). Germ cells normally go through two rounds of epigenetic DNA methylation erasure and re‐setting: one during primordial germ cell development while the embryonic germ cells are migrating to the genital ridge, and one at the time of fertilization when male and female pronuclei are coming together.^11^ It is hypothesized that one method by which epigenetic inheritance may occur is for certain DNA methylation patterns in gametes and the early embryo to escape this normal re‐setting, similar to what occurs for some imprinted genes. Other epigenetic factors may also be passed in sperm, such as non‐coding RNAs and retained histones.^12^ Recent epidemiological studies based on probabilistic models examined and supported intergenerational epigenetic inheritance,^13^, ^14^ where it was shown that at some CpG sites, the association of DNA methylation between mothers and offspring was stronger compared to the association between fathers and offspring. The number of CpGs with fathers being dominant in DNA methylation transmission is much smaller. Ancestral exposure to a variety of environmental insults such as abnormal nutrition, smoking exposure and behavioural stress has been shown to induce transgenerational inheritance of disease susceptibility including asthma. Epigenetic transgenerational inheritance has been demonstrated in plants, flies, worms, fish, rodents and pigs.^15^

### 3.2 Animal models of epigenetic inheritance

In a rat model, observed transgenerational epigenetic increases in susceptibility to diseases include increases in the incidence of germ cell apoptosis in testes, male infertility, kidney disease, prostate disease, polycystic ovarian disease, decreased ovarian follicles and increased rates of cancer.^16^ Transgenerational increases in rates of obesity and changes in behaviour have also been observed.^17^, ^18^

Several interesting findings have arisen from epigenetic transgenerational research in rats. Differential DNA methylation regions (DMRs; regions of the genome that are differentially methylated) between ancestrally toxicant‐exposed animals and controls are fewer in the F1 generation than in the transgenerational F3 generations, and the F1 generation DMRs are different than those of the F3 generation.^19^ Another interesting finding is that the pattern of DMRs in the transgenerational animals is specific to the ancestral toxicant exposure, suggesting that it may be possible to predict ancestral exposures in humans by examining epigenomic patterns in individuals.^15^ Also, most DMRs in rodent studies have been found in intergenic regions of low CpG density, rather than in CpG islands.^19^

### 3.3 Experimental transgenerational transmission of asthma risk after exposure to environmental particles

While studies in human cohorts are still underway to determine the association between epigenetic alterations and transmission of the phenotype, a major challenge is to determine whether aberrant DNA methylation can be causative in this transgenerational transmission. Hence, an experimental in vivo approach to track the aberrant methylation in three generations of mice whose increased asthma risk arises from a single ancestral exposure to environmental particles has been developed.

In this model, F1 offspring of female mice sensitized to ovalbumin allergen are predisposed to asthma development. The predisposition is allergen independent and transmits via the maternal not the paternal line,^20^ recreating similar observations in humans.^21^ This maternal transmission was associated with altered DNA methylation in dendritic cells (DCs).^22^, ^23^ Furthermore, adoptive transfer of DCs (but not other immune cells) from pups of asthmatic mothers to normal pups transfers the asthma predisposition, indicating that DCs have a central, causative role in establishing increased asthma risk.^22^ Exposure of pregnant mice to diesel exhaust particles or concentrated urban air particles acts similar to maternal asthma and results in a similar increase in asthma susceptibility in F1 pups, similarly mediated by DCs.^24^

This increased asthma susceptibility transmitted not only to F1 but also to F2 and to a lesser extent F3 generations (Figure 1). DCs in these generations continued to harbour aberrant methylation in over 400 loci including promoters of genes associated with lung development, IL‐4 signalling and chromatin dynamics. The number of affected loci diminishes from F1 to F2 and further to F3 generations, and some loci were found to be altered only in one but not in other generations.^25^

Finally, a subset of F1 dams were treated with a DNA methyl transferase inhibitor with the hypothesis that a ‘reshuffle’ of the epigenome could affect the transgenerational transmission of disease. Such treatment abrogated the transmission of asthma risk into F2 and F3 generations.^25^ It is worth noting that DNA methyl transferase inhibitors act broadly on the entire epigenome and have non‐epigenetic effects, highlighting the need for experimental ‘causality tools’ not yet available to study the role of epigenetic aberrations.

This finding is consistent with other human and experimental data suggesting that asthma risk can be transmitted transgenerationally from a single exposure to the deleterious factor and suggests that the nature of this transmission is in part via epigenetic (DNA methylation) changes in DCs.^25^, ^26^, ^27^

## 4 DEVELOPMENTAL PROGRAMMING AND EARLY LIFE ORIGINS OF DISEASE

The strongest evidence for the importance of developmental programming in allergic disease is the observation of marked phenotypic differences already apparent at birth between individuals who do, or do not, develop allergic conditions later in life.^28^ For example, children who develop asthma have impaired lung function shortly after birth in comparison to healthy children.^29^ Neonates with allergic predisposition also have recognized differences in many aspects of immune function at birth.^28^ While some of these differences will be due to inherited genetic predisposition to allergic disease, there is a clear role for maternal environmental exposures during pregnancy influencing subsequent development of allergy in offspring. For example, maternal allergy – and hence presumably an altered in utero environment – has an additional effect on risk of allergy in the offspring.^30^ Evidence for the in utero origins of asthma comes from associations of childhood asthma with maternal pre‐natal conditions and exposures such as maternal age, diet, smoking, infectious illness, stress, weight gain and exposure to farm animals.^21^, ^31^, ^32^ The detection of asthma‐associated differential epigenetic programming in neonates also supports the possibility that asthma might originate in utero.^33^, ^34^, ^35^

### 4.1 Maternal influences on childhood asthma risk

The maternal cytokine profile during the third trimester of pregnancy (defined as the ratio of IFN‐γ and IL‐13 secreted by mitogen‐stimulated maternal peripheral blood mononuclear cells) has an inverse association with childhood asthma risk. Interestingly, this relationship was independent of childhood allergy, was found for pre‐natal but not post‐natal maternal cytokine levels, was evident in children of non‐asthmatic but not in children of asthmatic mothers and held for maternal but not paternal cytokines.^36^ Ongoing work is currently investigating whether this relationship involves the neonatal epigenome. Unpublished data show that a substantial proportion of the *neonatal* differential methylation associated with the *maternal* cytokine ratio is also associated with *childhood* asthma, thereby pointing to an epigenetic trajectory that connects immune responses in the mother to asthma in her child.

### 4.2 Epigenetic predictors of childhood asthma at birth

Epigenetic studies focus on how a cell or a tissue become poised to express a given set of genes – that is, on a trajectory to events that will occur at a later time, thanks to epigenetic programming. The vast majority of epidemiological studies in humans on epigenetics in allergic disease have focussed on DNA methylation as it is more easily measured in comparison to other changes such as chromatin modifications, and it is chemically stable, making it possible to measure in stored tissue or DNA samples collected for other studies, e.g. genetics. Recent genome‐wide analysis of DNA methylation testified to the power of this approach by establishing a nexus between the immune epigenome at birth and asthma.^37^ In the Infant Immune Study, cord blood mononuclear cells of neonates who developed asthma by 9 years carried DNA methylation profiles distinct from those of neonates who did not become asthmatic.^38^ Some of these results were especially suggestive. Methylation of the *SMAD3* promoter, not only in Infant Immune Study neonates but also in two replication cohorts (the Manchester Asthma and Allergy Study and the Childhood Origins of ASThma study), was selectively and significantly increased in neonates born to asthmatic mothers and was associated with childhood asthma risk. These findings provided the first evidence that the trajectory to childhood asthma may begin at birth with epigenetic modifications that cluster primarily in the *SMAD3* pathway and are influenced by maternal asthma status. *SMAD3* is a well‐replicated asthma risk gene,^5^ and a master regulator of TGF‐β‐dependent signalling. In this capacity, *SMAD3* is uniquely positioned to affect the trajectory to, and the pathogenesis of, childhood asthma.^38^ Additionally, *SMAD3* and TGF‐β are expressed at high levels during early lung development regulating branching morphogenesis, epithelial cell differentiation and maturation of surfactant synthesis.^39^ The identification of *SMAD3* as an epigenetic predictor of childhood asthma at birth suggests that the early epigenetic trajectory to asthma proceeds at the intersection between immune regulation and lung development. This observation is supported by the pregnancy and childhood epigenetic consortium metanalysis in new‐borns (8 cohorts, 668 cases) identifying 9 CpGs (and 35 regions) differentially methylated (epigenome‐wide significance, false discovery rate <0.05) in relation to childhood wheeze.^33^ Lastly, consideration of genetic background further complicates investigations of environmental effects on epigenetics underlying potential intergenerational transmission of disease. Candidate gene studies have demonstrated that there are interaction effects between genotype and exposure on methylation, and genotype and methylation on outcomes.^40^, ^41^, ^42^, ^43^ Of note, a recent study explored the hypothesis that pre‐natal maternal immune dysfunction associated with increased childhood asthma risk (revealed by low IFN‐γ:IL‐13 secretion during the third trimester of pregnancy) alters immune training at birth through epigenetic mechanisms and promotes early‐life airway colonization by asthmagenic microbiota.^44^

## 5 TRANSGENERATIONAL INHERITANCE OF DISEASE SUSCEPTIBILITY

### 5.1 Studies of birth cohorts

Birth cohorts are ideal to investigate the impact of genetic predisposition, epigenetics and exposures during early life as data and samples are available before disease development. However, birth cohorts present significant challenges including recruitment and assessment over several decades, attrition over time and lack of generalizability if based in one geographical location. Collaboration between cohorts with formation of new consortia or using existing birth cohort alliances provides an opportunity to address these challenges.

**FIGURE 2:**
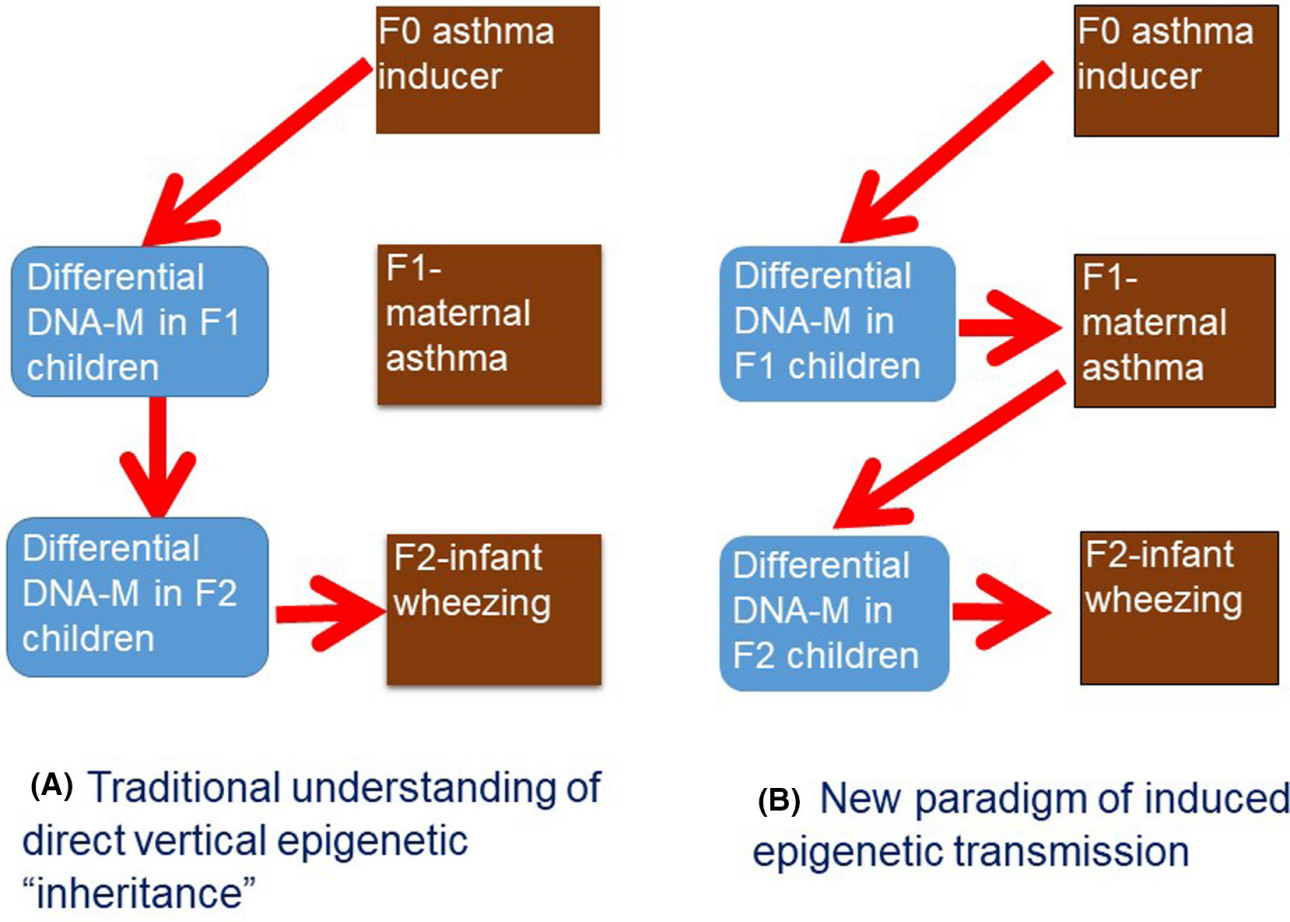
Vertical and induced epigenetic transmission models. (A) Vertical epigenetic transmission model proposes direct transfer of epigenetic information from parents to child in successive generations. (B) The alternative model, induced epigenetic transmission, proposes that maternal disease is an essential element in the chain of epigenetic transmission of information. DNA‐M, DNA methylation. Adapted from Arshad et al.^2^

The three generation Isle of Wight cohort study is the oldest prospective multi‐generation birth cohort with a focus on asthma, allergies and lung function.^45^ It has confirmed previous reports of the effect of grand maternal smoking during pregnancy on grandchildren's risk of wheezing.^2^ However, genome‐wide epigenotyping in parents and children did not confirm direct transfer of epigenetic markers. At a genome‐wide scale, <1% CpGs showed epigenetic associations between parents and their children. In addition, for most CpG sites that showed associations with the respective parental CpG sites, the associations were eliminated once the offspring genotype was taken into consideration (unpublished data). Thus, there is so far little evidence of a direct transfer of risk across multiple generations through epigenetic mechanisms. Although incomplete erasure of DNA methylation markers remains a potential explanation given the few CpGs that escape erasure might be relevant to disease risk, other explanations should be sought. An alternative explanation is that maternal conditions during pregnancy (rather than maternal epigenome) influence offspring epigenetics and subsequently risk of disease in the next generation (termed ‘induced epigenetic transmission’), resulting in a chain of transmission of epigenetics over generations, not requiring a transfer of epigenetic mark directly across generations. For instance, the explanation for grandmaternal asthma increasing the risk of grandchildren asthma is not epigenetic marks carrying the susceptibility risk of asthma vertically across the generations but rather grandmaternal asthma altering DNA methylation in her daughter and inducing asthma, who would do the same for her children, thus increasing the risk of asthma across generations without the need for vertical transfer of epigenetic markers (Figure 2). This hypothesis is supported by observation that maternal asthma influences epigenetics in offspring^38^ and improved management of maternal asthma during pregnancy results in reduced childhood asthma.^46^ Further, we have shown that maternal birth weight and BMI mediate the transgenerational effect of grand‐maternal BMI on grandchild's birth weight.^47^ Cumulatively, these observations support a role of epigenetics as an underlying mechanism in transfer of disease risk across generations.

**FIGURE 3:**
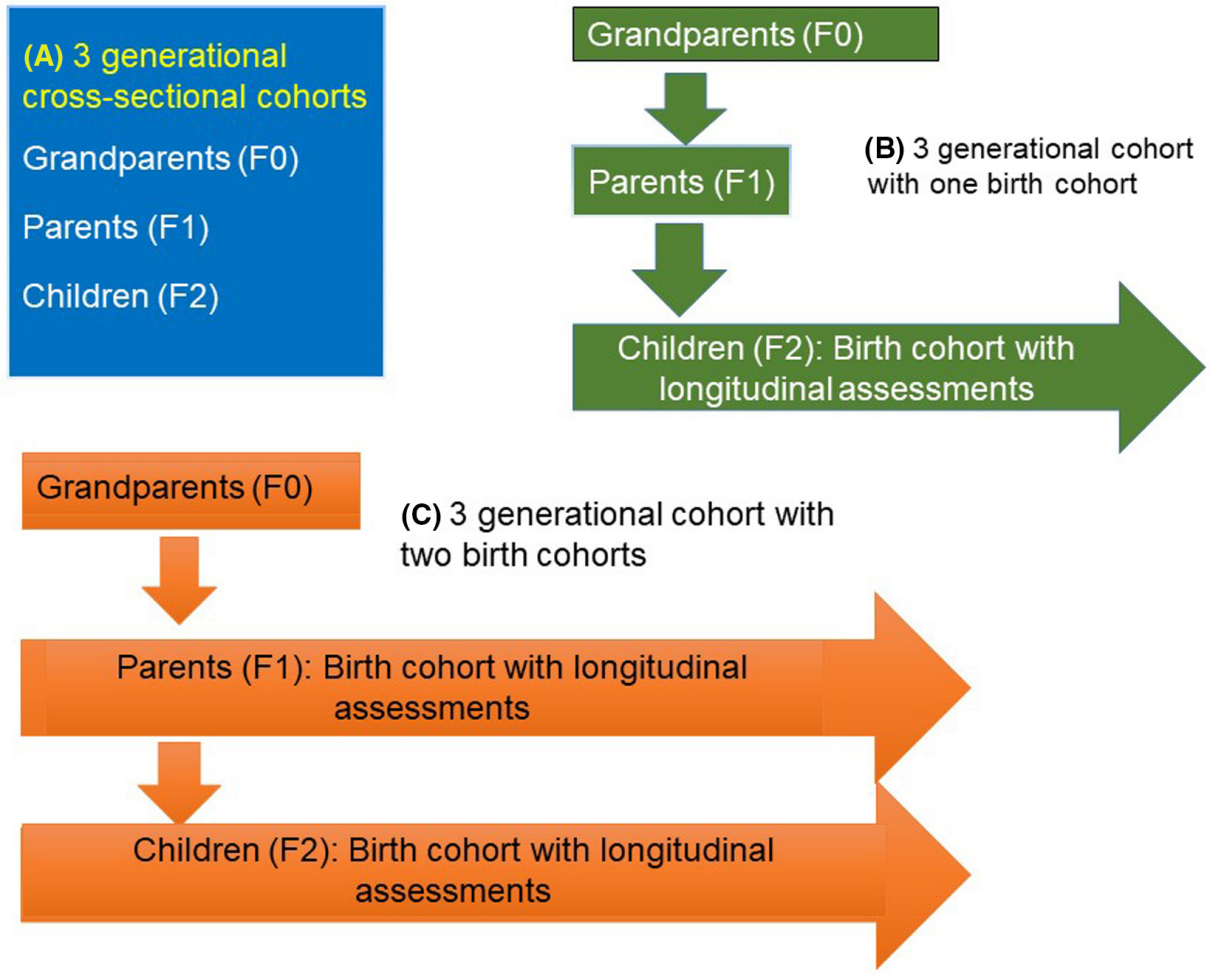
Models of multi‐generation cohorts. (A) In three generational cross‐sectional cohorts, grandparents (F0) and parents (F1) are recruited in their adult life while grandchildren (F2) could be children or adult. They may be assessed once or followed up longitudinally. (B) Some three generational cohorts include a birth cohort, where two generations (grandparents and parents) are recruited in adult life, while grandchildren are recruited at birth and followed up longitudinally. (C) Transgenerational cohorts will have at least two birth cohorts so that both F1 and F2 are recruited at birth.

Several other multi‐generational studies include one or two birth cohorts (Figure 3). The Lifeways cross‐generation cohort study recruited over 1000 children at birth as well as parents and grandparents of both lineages with a focus on wheeze and obesity.^48^ In Germany, children born to participants of the International Study of Asthma and Allergies in Childhood are being recruited in a multi‐generational study with information also collected from the parents of the original cohort and DNA collected for epigenetic analysis (ACROSSOLAR study).^49^ In the UK, the ALSPAC study is recruiting both the parents and the offspring of the original birth cohort, thus providing information on three generations, two being the birth cohorts.^50^ Asthma, allergy and obesity are (among others) the disease foci and both genetic and epigenetic mechanisms are being studied. In California, the Children's Health Study (CHS) originally recruited school‐age children and their parents for a study of the respiratory health effects of air pollution.^51^, ^52^ CHS subjects, now grown, are being recontacted and their own children recruited into a follow‐up study to evaluate epigenetics across three generations. Data from these studies will enhance our knowledge of the extent of transgenerational effects.

### 5.2 Grandparental smoking patterns associated with childhood asthma

A wealth of data exists illustrating the adverse health effects of smoking during pregnancy on the fetus and developing child.^53^, ^54^, ^55^ Evidence for effects of grandparental smoking on child health is also emerging (Table 1).^56^ Recent publications suggest grandmother's smoking may increase risk of asthma in the child independent of whether either parent smoked.^57^, ^58^, ^59^, ^60^ Father's smoking at young ages may also increase risk of asthma and low lung function in their future children.^4^, ^58^, ^61^

**TABLE 1:** Influence of maternal and grandmaternal smoking during pregnancy on early childhood wheeze

	Odds ratio; 95% CI
No maternal or grandmaternal smoking	Reference
Only maternal smoking	0.9, CI: 0.4–2.3
Only grandmaternal smoking	0.9, CI: 0.3–2.4
Maternal and grandmaternal smoking	2.6, CI: 0.9–7.1

*Note*: Data from the Isle of Wight 3rd Generation cohort.

One mechanism that can help explain inherited risk across generations is an altered epigenome in response to pollutant exposures.^62^ Several epigenetic models of inheritance have been proposed in support of this hypothesis and are discussed below though many aspects of these models are as yet untested.^2^ To date, numerous human epigenome‐wide studies of maternal smoking effects on DNA methylation in the new‐born have found evidence for hundreds if not thousands of loci potentially affected by exposure.^63^, ^64^, ^65^ Moreover, many identified methylation loci are known to have roles in asthma development or lung function growth, suggesting a potential mediating role for DNA methylation. For example, a meta‐analysis from the PACE consortium found that 59 DMRs were associated with childhood lung function, of which 18 were associated with childhood asthma and nine with COPD in adulthood.^66^ In another study, pre‐natal tobacco smoke was associated with higher methylation level in the AXL gene in two distinct cohorts, and the combination of higher AXL methylation and PTS exposure at birth increased the risk of recent episodes of bronchitis symptoms in childhood.^65^ Further to this, recent studies have demonstrated effects of grandmaternal smoking on grandchildren's DNA methylation at a small number of CpG sites.^67^, ^68^

### 5.3 Multi‐generation studies based on adult cohorts

There are very few three‐generation epidemiological studies reported in the literature. The Panel Study of Income Dynamics (PSID) involving some 8000 US families linked with the Child Development Supplement of 2900 children within PSID families allowed interview of multiple generations in the same family.^69^ Compared with the reference group in which no parent or grandparent had asthma, the risk for childhood asthma if a parent had asthma was OR 1.96 (95% CI 1.26–3.05); if only a grandparent had asthma, the OR was 1.52 (95% CI 0.98–2.35), while the risk was greatest if both a parent and a grandparent had asthma, OR 4.27 (95% CI 2.39–7.65).

The RHINESSA study [Respiratory Health In Northern Europe, Spain and Australia, (www.rhinessa.net)] is designed to investigate the influence of exposures before conception and in previous generations on allergies and lung health; to identify windows of susceptibility to environmental influence operating before and after conception and to explore mechanisms for exposure effects across generations.^70^ The RHINESSA study investigates the offspring (G2) of persons who have already been extensively characterized during 20 years of their reproductive life (G1) in two large studies: the population‐based cohorts ECRHS (European Community Respiratory Health Survey, clinical data, www.ecrhs.org) established in the early 1990s and the linked study RHINE (Respiratory Health in Northern Europe) (questionnaire data, www.rhine.nu).

### 5.4 The importance of puberty in men on risk in offspring

Analyses of the RHINESSA/RHINE/ECRHS cohorts suggest, consistently, that father's exposures in early puberty may be of key importance for asthma and lung function in the offspring.^4^, ^58^, ^70^, ^71^ In the RHINESSA cohort, father's smoking before age 15 years was strongly and consistently associated with early‐onset offspring's non‐allergic asthma, while duration of smoking, but not time of quitting, was of some importance. Further, a doubled risk of asthma was found for offspring of fathers with an occupational history of welding for at least 10 years before conception. Asthma was further associated with paternal grandmother's smoking – which, curiously, significantly modified the associations with father's smoking, totally attenuating effects of father's early onset smoking, but enhancing effects of father's later onset smoking or welding. Association of father's smoking <15 years was replicated in a multi‐generation analysis of the ECRHS cohort. Statistical methods were developed to account for the complexity in multi‐centre multi‐generation data, including simulation models that found that the estimated effect of unaccounted‐for confounding was low.^72^ Using more advanced statistical models for causal inference from observational data, father's smoking before age 15 years was a causal factor also for low lung function in offspring.^61^ This was found both for FEV_1_ and for FVC, suggesting effects on lung growth as well as airways calibre.

Further support for an important susceptibility window in early male puberty was obtained by a study of parents' puberty onset overweight. Johannessen et al. found that father's becoming overweight before voice break but after age 8 years was causally related to asthma in future offspring.^70^ This effect was not mediated by offspring's own weight, nor were there significant effects of father's being overweight after puberty or mother's being overweight before conception. A recent study found that father's prepuberty onset overweight also was associated with lower lung function in offspring, and with considerably lower adult height in sons.^73^ These observations have been supported by findings in Tasmanian Longitudinal Health Study showing association between paternal BMI trajectories from childhood to adolescence and asthma in their future offspring.^74^ These analyses consistently suggest that there are significant windows of environmental exposure, such as male puberty and prior to conception that may be important with regard to offspring respiratory health. It seems plausible that male puberty could be a time window of higher susceptibility to environmental exposures due to epigenetic reprogramming during that period.^10^, ^75^

In addition to these studies that point to a role of the father's preconception environment, in particular during prepuberty, there are studies pointing to the mother's preconception.^76^ A recent study identified higher asthma risk in offspring associated with mother's occupational exposure to cleaning products and disinfectants.^27^

## 6 DATA ANALYSIS IN EPIGENETIC RESEARCH AND CRITICAL REVIEW OF DATA

Statistical analysis of multi‐generation data poses challenges, including varying degree of longitudinal and hierarchical data, and the need to test multiple pathways and complex theoretical models. Path analyses, structural equation modelling and multilevel modelling are all relevant in this context. Accordini et al. have developed models to meet some of these challenges.^61^ Data analyses in transgenerational epigenetic research mainly focus on DNA methylation transmission from one generation to the next and transgenerational association studies.^13^, ^14^, ^67^, ^68^ Further development of multiple‐exposure, multiple‐mediator and multiple‐outcome framework in studies of multi‐generational epigenetic inheritance is needed.

Unlike assessment in genetic inheritance, evaluation of epigenetic inheritance intergenerationally faces challenges in multi‐ways. Correlation‐ and intra‐class correlation coefficient‐based methods assess transmission or inheritance at the individual level. However, agreement at the individual level does not guarantee agreement at the population level.^13^ Furthermore, to our knowledge, all currently available analytical methods for DNA methylation transmission between generations are based on numerical similarity. However, such similarity does not have to be the consequence of transmission or inheritance. Biological mechanisms or markers of inheritance need to be incorporated into the numerical assessment of strength of DNA methylation transmission or inheritance.

It has been acknowledged that DNA methylation changes with chronological ageing. To evaluate DNA methylation changes due to exposure, health conditions and other substantial events in life such as puberty events, it is critical to tease out the changes due to ageing. This is not addressed in most existing longitudinal studies and the confounding effects of chronological ageing need a thorough consideration in future investigations. In intergenerational and transgenerational studies, adjusting chronological ageing, however, may not be so critical, as long as no longitudinal DNA methylation within a generation is under investigation, since associations between DNA methylation, rather than changes in DNA methylation, and end points in different generations are the main focus. For instance, in studies assessing the role of DNA methylation as mediators between generations,^77^, ^78^, ^79^ structural equation modelling is commonly applied, in which case one time, e.g., at birth, DNA methylation at a set of CpG sites in a generation is included as potential mediators.

Methods to address joint effects of a set of CpG sites have been proposed, e.g., detection of DMRs,^80^ high‐dimensional mediation analyses on a set of methylation sites or epigenetic network studies. However, some of these methods do not address inter‐connections between CpG sites such as methods for DMRs and those to assess mediation effects of a set of CpG sites.^77^, ^78^, ^79^ Some do not incorporate biological pathways, e.g., epigenetic networks constructed based on associations among CpG sites^81^, ^82^ or correlation networks^83^ based on pairwise correlations between CpGs in DNA methylation. Because of these, inferences may be incomplete, biased or even misleading. Directed networks, e.g., via directed acyclic graphs, among CpGs have the potential to assess causality between epigenetic sites, but methods with the ability to address epigenetic changes over time are in great need.

As with all genome‐scale data, DNA methylation data need to go through pipelines of quality control and pre‐processing. Although multiple methods have been proposed, all these approaches in general involve a combination of quantile normalization of raw DNA methylation data followed by approaches to remove batch effects and technical variations.^84^, ^85^, ^86^, ^87^ In some situations, these pre‐processing methods are not able to fully remove batch effects or technical variation. In such cases, surrogate variables are usually estimated and included in analyses. Surrogate variables are latent variables representing unknown factors that potentially affect the quality of DNA methylation.

An issue closely related to DNA methylation pre‐processing is the heterogeneity of cell types within samples. For example, DNA methylation is usually measured in whole peripheral blood samples and is influenced by heterogeneity of cell compositions in the blood. Methods have been proposed to infer cell type proportions,^85^, ^88^, ^89^ which are then included in data analyses to adjust for the effects of cell heterogeneity. However, these methods are sensitive to the selected reference database used as the standard.^90^ Methods not relying on reference databases, mostly based on principal component analyses or factor analyses, have been proposed.^89^ Novel methods with the ability to infer cell type proportions, which are robust against the effect related to the choice of reference databases, are greatly needed. This variation in epigenetic factors among cells and tissues as a consequence of cellular differentiation is a major limitation of current epigenetic epidemiological studies. Usually blood or buccal/saliva DNA is used for assessment of epigenetic profiles due to ease of sampling. However, it is still unclear to what extent pre‐conceptional or early‐life exposure associated differences in methylation in blood are also observable in other disease relevant tissue, e.g., lung.

To improve statistical power in genome‐scale studies, screening of methylation sites is usually performed prior to data analyses. Statistical approaches have been proposed to filter out irrelevant or non‐informative CpG sites.^91^, ^92^ To avoid data double dipping, statistical models for screening should be different from the models to be applied in final analyses (Box 1).

BOX 1 **Recommendations and challenges**

- New multi‐generational studies in humans are needed, specifically, well‐characterized cohorts with longitudinal information to avoid confounding by disease and treatment effects and recall bias. The challenge is to recruit and maintain such cohorts over decades, with complex study designs and costly recording and analysis of time‐dependent exposures. Collaboration between cohorts is desired to achieve the required sample size to generate robust evidence.
- Family based transgenerational studies will have the power to assess within family factors such as birth order and environmental exposures shared within family, as well as between family factors.
- Methylation markers in the blood may need further validation in relevant tissues such as nose or lung for the study of rhinitis and asthma/lung function, respectively.
- Future studies should also investigate the functional consequences of these epigenetic signatures, transferred across generations, which emphasized the unmet needs for novel epigenetic editing tools.
- A challenge in statistical methods to examine epigenetic effects in transgenerational or intergenerational studies is to properly incorporate underlying biological mechanisms into the design of analytical methods.
- An important question to ask is whether it is possible to intervene to modify the epigenome with post‐natal exposures (treatments) to prevent disease?
- These knowledge gaps will be important to address in future studies as we seek to better understand the risk factors for asthma and allergic disease and devise optimal public health strategies to prevent these chronic conditions to improve the health of the population.

## 7 SUMMARY

Recent evidence indicates that grand parental health status, nutrition and environmental exposures might influence health outcomes in grandchildren. Therefore, the risk to individual's health should consider not only parental genetics and exposures but also grandparental and ancestral factors. While researchers have begun to systematically investigate DNA methylation, we have barely scratched the surface in the investigations of other epigenetic factors. Timing of exposures, biologically relevant tissues in which to measure epigenomics, and genetic background are important considerations. Cross‐generational studies can provide useful insights on transgenerational effect, especially where preconception exposure information is available.

## AUTHOR CONTRIBUTIONS

This review is based on the proceedings of a workshop organized by the National Institute of Allergy and Infectious Diseases. All authors were participants of the workshop and they contributed to the first draft by writing sections based on their presentations at the workshop. These sections were compiled by Lisa Wheatley to produce the first draft of the manuscript. Hasan Arshad, who organized the workshop with assistance from Lisa Wheatley and Alkis Togias, revised the draft to convert it into a narrative review. All authors then reviewed, revised and approved the manuscript.

## CONFLICT OF INTEREST

The authors declare that they have no conflict of interest with regard to this publication.

## Data Availability

The data that support the findings of this study are available on request from the corresponding author. The data are not publicly available due to privacy or ethical restrictions.
